# Differential Expression of the Androgen Receptor, Splice Variants and Relaxin 2 in Renal Cancer

**DOI:** 10.3390/life11080731

**Published:** 2021-07-22

**Authors:** Joanna Bialek, Maria Piwonka, Felix Kawan, Paolo Fornara, Gerit Theil

**Affiliations:** Medical Faculty of Martin Luther University Halle-Wittenberg, University Clinic and Outpatient Clinic for Urology, 06120 Halle (Saale), Germany; maria.piwonka@uk-halle.de (M.P.); felix.kawan@uk-halle.de (F.K.); paolo.fornara@uk-halle.de (P.F.); gerit.theil@uk-halle.de (G.T.)

**Keywords:** renal carcinoma, ccRCC, pRCC, androgen receptor, AR, AR splice variants, relaxin, RLN2

## Abstract

Background: The role of the androgen receptor (AR) in renal cell carcinoma (RCC) is unclear. We aimed to analyze the expression of AR and its splice variants (SVs) and their correlation with relaxin 2 (RLN2) and cytokines in RCC. Methods: We investigated the expression of RLN2 and AR variants in 25 clear cell RCC (ccRCC) and 9 papillary (pRCC) tumor tissues and the corresponding controls using quantitative PCR and serum RLN2, testosterone and cytokine levels in matched samples using ELISA and chemiluminescent immunometric assay, respectively. Results: ccRCC tissues but not pRCC tissues more frequently expressed AR and the SVs than did normal tissues. All pRCC samples expressed more AR than did ccRCC samples. The highest expression of all AR variants except AR-V12 was found in low-stage tumors, with dominant expression of AR-V7. In males in the ccRCC cohort, the expression of AR-FL, AR-V1 and AR-V3 was significantly correlated with that of RLN2. The secretion pattern of proinflammatory IL-6 was higher in ccRCC than in pRCC. Conclusions: The results highlight additional molecular differences between ccRCC and pRCC, suggesting the influence of external factors on the whole kidney or genetic predispositions to developing certain types of renal cancer, and may support further pathological analysis and studies of targeted hormone therapy.

## 1. Introduction

Renal cancer is one of the most common cancers worldwide, reaching over 403,000 new cases in 2018 and representing 2.2% of newly diagnosed cancers [1,2]. Approximately 15% of patients already demonstrate metastases by the time of initial presentation. This is one of the factors that contribute to the high mortality rate (>175,000 deaths per year) of renal cell carcinoma (RCC) among the urological malignancies [2].

The two most common RCC subtypes are clear cell RCC (ccRCC) and papillary RCC (pRCC). The first type, ccRCC, accounts for 75% of all RCCs, while pRCC (subtype 1 and 2) accounts for approximately 10%. Both tumors differ in vascularization. Clear cell RCC is highly vascularized, while pRCCs are hypovascularized when compared to the surrounding parenchyma [3,4]. The ccRCC patients have a worse prognosis than pRCC patients and approximately 15% of them develop lung, liver, bone or lymph node metastasis [4].

According to clinical observations, the incidence of RCC is twice as high in men as in women, which suggests the involvement of steroid hormone receptors in tumor development. Expression analyses often reveal relatively high levels of androgen receptors (AR) in normal kidneys and lower levels in tumorous kidneys. Several reports have described that AR expression tends to decrease with increasing pT stage and Fuhrman’s grade, while others have presented contradictory results. Moreover, primary tumors display higher expression of AR than metastases [5,6,7]. Such data were also described in online databases. According to Protein Atlas, AR, which is detected in renal epithelial cells, is considered a favorable prognostic marker in RCC. Analyses performed with 877 patients showed that high expression of AR (*n* = 571) was associated with longer survival probability (at least 16 years), while lower expression (*n* = 306) was associated with shorter survival probability (13 years). Patients in this collective were not analyzed with regard to sex or age [8]. Analysis carried out in GENT2 showed no differences between AR expression in normal and tumor tissues [9]. A graphical summary of these data is included in Supplementary Figure S1. Analysis of signal transduction in castration-resistant prostate carcinoma (CRPC) has indicated the role of AR in the activation of genes involved in metabolism, secretion and differentiation [10], although the meaning of the AR in RCC is still not clear.

After hormone binding and dimerization of monomers, the AR translocates to the nucleus and initiates activation of target genes. This mechanism is well studied in prostate cancer. This acquired knowledge was fundamental in developing AR target therapy and medicines for prostate cancer patients [11].

In addition to its analysis in prostate cancer, AR expression has been analyzed in other tumors, such as bladder [12], breast [13], pancreas [14], liver [15] and ovary [16] tumors, providing additional information about tumor growth, survival time (liver, bladder [12]) and improvements for antiandrogen therapy (breast [17]).

Target therapies are also administered to advanced or metastatic RCC patients. These therapies target, for example, vascular endothelial growth factor (VEGF) and tyrosine kinase inhibitors (TKIs) [18]; however, due to adaptation of the tumor microenvironment, resistance may occur [19]. For this reason, molecular analysis of RCC and a better understanding of the disease are crucial to increase the potential of personalized treatment for these patients. Recently, the administration of immune checkpoint inhibitors such as PD-1/PD-L1 to ccRCC and pRCC patients has shown positive improvements in overall survival [18].

Detecting the splice variants (SVs) of AR and their constitutive expression and functionality has increased the significance of AR in terms of pathogenesis. Over 30 variants have been identified to date [20]. Most of these lack parts of the ligand-binding domain (LBD), which is the target of enzalutamide, but contain cryptic exon fragments [21]. The most commonly studied SV is AR-V7. In prostate carcinoma (PCa), the expression of constitutively active AR-V7 is correlated with resistance to androgen deprivation therapy (ADT) [20]. The role of AR-V7 as a biomarker expressed in the circulating tumor cells (CTCs) of PCa patients was discussed by Theil et al. [22], among others. Until now, outside of the prostate, its expression has been detected in breast tissue [23,24]. The other most abundant variant in PCa is AR-V1, which is increased in CRPC in comparison to hormone-naïve bone metastases [21]. According to Lu et al. [25], three more constitutively expressed splice variants exist: AR-V3, AR-V4 and AR-V12 (ARv567es); however, it is still debated whether AR-V12 is created as the result of alternative splicing or gene rearrangement [26].

The androgen receptor can be activated in an androgen-independent manner. One of the proteins that influence the AR signaling pathway is relaxin 2 (RLN2) [27]. RLN2 is a small, 6 kDa hormone that is involved in physiological and pathological conditions. Its activity during pregnancy and its involvement in several tumors have been widely described [27,28,29,30,31,32], although the role of RLN2 in RCC is not well understood.

The physiological expression of relaxin (and its receptor RXFP1) in the kidneys is not high. Even if its functional significance is still not clear, its protective activity has been suggested [32]. In particular, endogenous RLN2 is considered to serve as a renoprotective factor against fibrosis in the aging kidneys or after injury; however, this activity is sex-specific [33,34]. Moreover, in early tubulointerstitial renal disease, RLN2 inhibits the differentiation of renal myofibroblasts, which in turn are probably unable to synthesize aberrant collagen related to renal fibrosis [33,35]. Additionally, a protective role of RLN2 was noticed in the promotion of renal vasodilation and hyperfiltration [33,36], as well as angiogenesis [37].

Several reports indicate that the expression and activity of AR and RLN2 correlate with the synthesis of proinflammatory interleukins IL-6 and IL-8 under physiological and pathological conditions [38,39,40,41]. Parihar and Tunuguntla [42] demonstrated higher expression of IL-8 in metastatic RCC, additionally suggesting IL-8 as a distinguishing marker for RCC. IL-8 expression was significantly higher in ccRCC than in oncocytic specimens [42,43]. With regard to IL-6, its presence in the serum together with elevated soluble intercellular adhesion molecule-1 (sICAM-1) levels was found to be related to unfavorable prognosis in RCC [44]. In vitro analysis has revealed the involvement of IL-6 in the tumor invasion process [45].

In our studies, we investigated the expression of AR and AR-SVs and their correlation with RLN2 expression in both ccRCC and pRCC. Additionally, matched serum samples were investigated for the levels of the AR ligand- testosterone, RLN2 and two cytokines, IL-6 and IL-8.

## 2. Materials and Methods

### 2.1. Tissues and Serum Samples

We analyzed tumor tissues and corresponding tumor-free tissues (defined as “normal”) obtained from patients diagnosed with RCC that underwent nephrectomy in our clinic. The tumor tissues collected from treatment-naïve patients were of different types (ccRCC *n* = 25; pRCC *n* = 9; papillary adenoma *n* = 1) and stages (pT1–pT4) (Table 1), and the matched serum samples were collected in 4.5 mL tubes. All patients provided written informed consent. The medical faculty ethics committee of Martin Luther University Halle-Wittenberg approved the study protocol (2012-65).

### 2.2. Cell Lines

The renal carcinoma cell line Caki-1 (ATCC, Manassas, USA) was grown in RPMI medium (Life Technologies, Darmstadt, Germany) and the PCa cell line LNCaP (ATCC)-was grown in DMEM enriched with 10% FCS (Capricorn Scientific GmbH, Ebsdorfergrund, Germany). The medium was changed every 2–3 days and both cell lines were passaged every 4–5 days.

### 2.3. RNA Isolation and Quantitative RT-PCR

Total RNA was isolated from homogenized frozen tissues using an RNeasy Plus Mini Kit (Qiagen, Hilden, Germany) according to the manufacturer’s instructions. Subsequently, cDNA was synthetized from 500 µg RNA with SuperScript IV VILO Master Mix (Thermo Fisher Scientific, Aahen, Germany). Quantitative PCR (qPCR) was performed using 5× Hot FirePol Eva Green qPCR Mix Plus (Solis Biodyne, Tartu, Estonia) with a QuantStudio5 Thermocycler (Thermo Fisher Scientific). Expression of the target genes was analyzed using specific primers, and β-actin served as endogen control (Table 2). The results were calculated with the 2^−ΔΔCT^ method.

### 2.4. Serum Protein Analysis

We analyzed the presence of two cytokines (IL-6 and IL-8) and testosterone in the serum of 25 ccRCC and 9 pRCC patients using Solid-Phase Chemiluminescent Immunometric Assay kits with an Immulite 1000 Immunoassay System and ADVIA Centaur CP (all Siemens Healthcare GmbH, Erlangen, Germany) according to manufacturer’s instructions.

Serum levels of RLN2 were analyzed with a Human Relaxin-2 Quantikine ELISA Kit (R&D Systems, Abingdon, United Kingdom) according to manufacturer’s instructions. Assay Diluent was mixed with the standard, control or samples in a provided 96-well plate and incubated for 2 hrs at RT. After washing, the Human Relaxin-2 Conjugate was added to each well and incubated for 2 hrs at RT. Following repeated washing and 30 min incubation with Substrate Solution, the reaction was stopped with Stop Solution and the optical density was determined using an Infinite M Plex microplate reader (Tecan Deutschland GmbH, Crailsheim, Germany).

### 2.5. Statistical Analyses

Prism 9 (GraphPad Software, La Jolla, CA, USA) was used to perform statistical analyses and produce figures. All data were tested for normal distribution (Shapiro–Wilk test). The data are presented as the median ± range, and possible correlations between different markers for the different tumor types were identified with Spearman’s rank test. Associations between the expression of AR (all variants) and other markers were tested using the Mann–Whitney test. Reported *p*-values are two-sided, and *p* ≤ 0.05 was considered to indicate significance.

## 3. Results

### 3.1. Androgen Receptor (AR) in RCC

**Figure 1:**
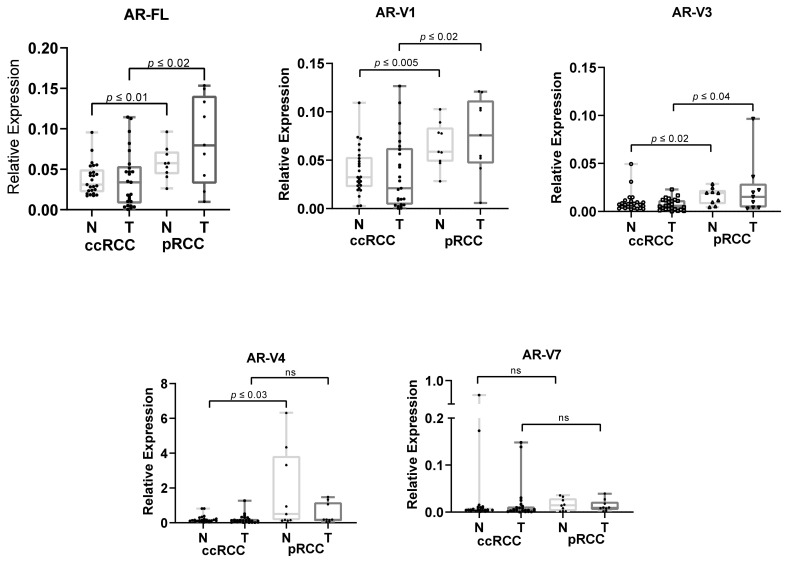
Relative expression of AR-FL and the SVs in tumor (T) and corresponding normal (N) tissues of ccRCC (*n* = 25) and *p*RCC (*n* = 9) displayed as median values normalized to the expression of the positive control (LNCaP). Differences were measured with a two-tailed Mann–Whitney test.

To clarify the significance of AR in renal carcinoma patients, 34 RCC tumors (ccRCC *n* = 25, pRCC *n* = 9), one papillary adenoma and corresponding nontumor tissues were subjected to qPCR analyses. The median age of the ccRCC patients was 67.5 years old (70—males, 64—females) and that of the pRCC patients was 70 years old (68—males, 74.5—females). Our transcriptome studies revealed expression of the full-length AR (AR-FL) as well as four of five tested SVs in normal and tumor tissue. The transcript variant AR-V12 was not detected in any sample (Figure 1).

We investigated the AR expression in terms of single SVs or single patients. In our cohorts, both normal and tumor tissues of pRCC patients had significantly higher expression of AR-FL (normal = 2 times higher, tumor = 2.7 times higher), AR-V1 (normal = 2 times higher, tumor = 3.5 times higher) and AR-V3 (normal = 2 times higher, tumor = 1.7 times higher) than normal and tumor tissues of ccRCC patients, as well as significantly higher expression of AR-V4 (normal = 5 times higher) than normal tissues of ccRCC patients. No significant difference between tumor and normal tissues of any type of RCC was detected (Figure 1).

**Figure 2:**
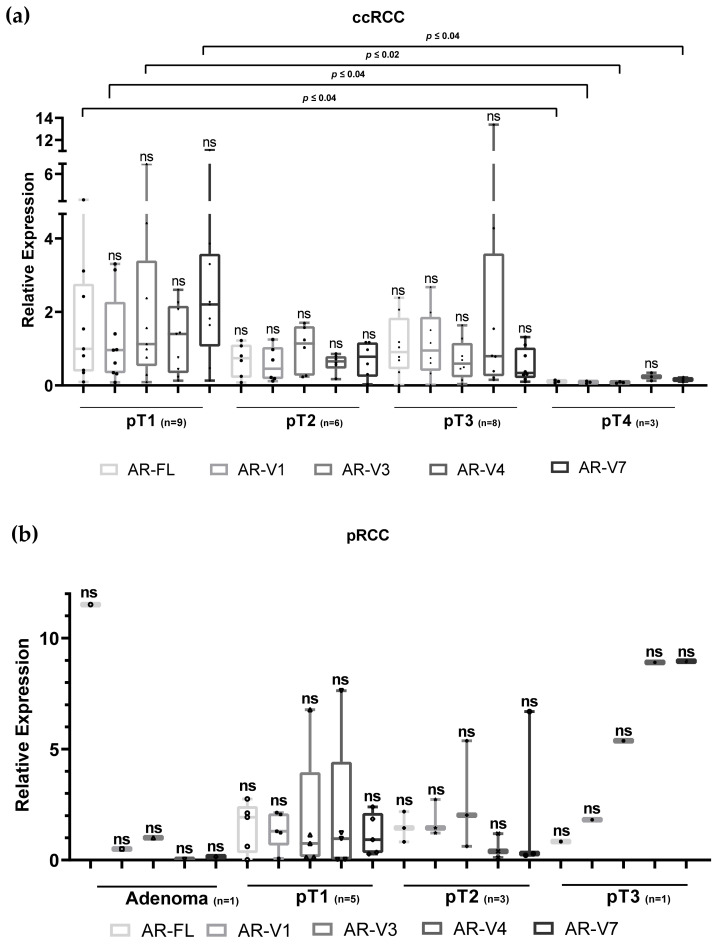
Relative expression of AR variants in ccRCC (**a**) and pRCC (**b**) tumors of different stages displayed as median values normalized to normal tissue values. Significant differences measured with a two-tailed Mann–Whitney test were noted only between pT1 and pT4 ccRCC tumor tissues (**a**). No significant differences were noted within the papillary group (**b**); *n*—number of samples.

We normalized the results by defining the values in corresponding control tissues as “1” and compared them to the values in the tumors. All tested variants (AR-FL, AR-V1, AR-V3, AR-V4 and AR-V7) displayed similar expression patterns, showing rarer expression in ccRCC tumors than in control tissue (normal expression > tumor expression in 56%, 56%, 52%, 64% and 48%, respectively) (Table 3). The average and median values showed the highest expression of almost all variants of the AR receptor (except the average of AR-V4 value) in small tumors (pT1) and the lowest expression in large tumors (pT4) (Figure 2). The highest difference among the ccRCC tissues was detected for AR-V3 (14.12 times; *p ≤* 0.02), followed by AR-V7 (13.75 times; *p ≤* 0.04) and AR-V1 (11 times; *p ≤* 0.04). The lowest difference in the same group was noted for AR-FL (10 times; *p ≤* 0.04) (Figure 2). Among all the variants, the strongest signal was detected for AR-V7, which was almost twice as strong as that of AR-FL (Figure 2). In regard to the patient-specific analysis (ccRCC), five patients had lower and three of them had higher AR expression in tumors, independent of the AR variant. There was no correlation between patient characteristics (male/female or age) and low or high levels of AR. Unlike in the ccRCC cohort, in the pRCC cohort AR-FL, AR-V1 and AR-V3 had more frequent expression in tumors (tumor expression > normal expression in 56%, 89%, and 56% of samples, respectively), while AR-V4 and AR-V7 expression dominated in control tissues (tumor expression < normal expression in 56% of samples for both variants) (Table 3). Analysis according to tumor size (pT1, pT2) revealed no significant differences. Papillary adenoma revealed much higher expression of AR-FL than other SVs (AR-V1: 23 times; AR-V3: 11.5 times; AR-V4: 575 times; AR-V7: 76 times), while in pT3 tissue, the results show that AR-FL expression was lower than that of other SVs (AR-V1: 2.5 times; AR-V3: 6.75 times; AR-V4: 11.12 times; AR-V7: 11.25 times). These are interesting results; however, they could not be statistically analyzed because of the limited number of samples. All ccRCC patients with higher expression of all AR variants in tumors had pT1 ccRCC, and all pT1 or pT2 pRCC (Table 3, Figure 2).

**Figure 3:**
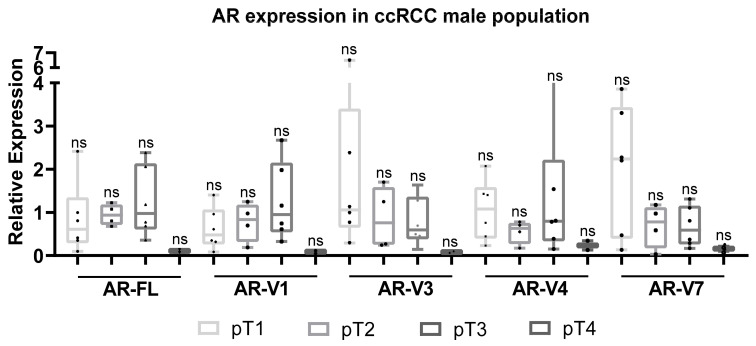
Analysis of AR variant expression in tumors of different stages in the male population of the cohort. The relative median expression values of the AR variants in different pT stages are shown.

Comparison of the male and female groups among the ccRCCs cohort did not show any significant differences. Analysis of the male patient group revealed slightly higher expression of AR-FL and AR-V1 in pT2 (median 0.94 (range 0.19–1.25) and 0.98 (range 0.24–1.7), respectively) and pT3 tumors (median 1.24 (range 0.32–2.67) and 0.83 (range 0.14–1.63) respectively), while the expression of AR-V3, AR-V4 and AR-V7 dominated in pT1 tumors (median 1.06 (range 0,76–6.49), 1.08 (range 0.23–2.07), 2.24 (range 0.47–3.86)) (Figure 3). It must be noted that even if the median expression of AR-V4 reaches the highest level in pT1 tumors (1.08), the most extensive range (0.15–4.27) was noticed in pT3 samples.

## 3.2. Association Between Expression of AR and RLN2 and RXFP1 Receptor

**Figure 4:**
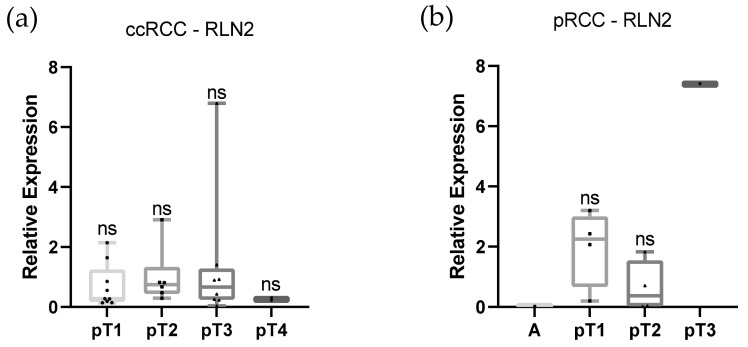
Expression of RLN2 in ccRCC (**a**) and pRCC (**b**) displayed as median values in tumors of different stages normalized to the expression in the Caki-1 cell line as positive control. Whiskers represent the minimum and maximum values. Differences were measured with a two-tailed Mann–Whitney test. “A”—adenoma.

We analyzed the expression of RLN2 mRNA in ccRCC and pRCC (Figure 4). In our cohort, the median RLN2 expression values of tumors of each size were lower than those in normal tissues. Higher expression was only observed in small (pT1) pRCC tumors. No statistically significant differences were noted between pT statuses.

Furthermore, we normalized the RLN2 expression values of both RCC subtypes, by defining the normal tissue values as “1” analyzed expression in different pT stages and compared the male and female populations (ccRCC). We did not find any significant differences between these groups. Results of the whole group of pRCC samples display alternations in mean (pT2 1.97 vs. pT3 0.64) and median (pT2 2.25 (range 0.2–3.2) vs. pT3 0.37 (0.01–1.82)) values; however, the low number of samples does not allow us to state the significant differences. The papillary adenoma and pT3 tumor stage samples of pRCC were statistically not analyzed because of the limited number of samples (Figure 4). No significant differences in expression of RLN2 between ccRCC and pRCC were noted (pT1 *p* ≤ 0.1, pT2 *p* ≤ 0.48)

Examination of the group of male patients revealed a significant positive correlation between expression of AR-FL and RLN2 expression (r ≤ 0.67; * *p* ≤ 0.002), AR-V1 and RLN2 expression (r ≤ 0.58; * *p* ≤ 0.01) and AR-V4 and RLN2 expression (r ≤ 0.49; * *p* ≤ 0.04) (Table 4).

**Figure 5:**
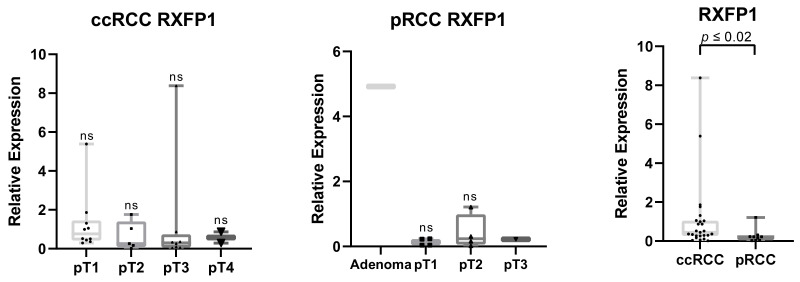
Expression of relaxin receptor RXFP1 in tumors. No significant differences were noted between tumor status within each tested group. Significantly higher expression was observed in ccRCC when compared to pRCC (*p* ≤ 0.02).

Additionally, we analyzed the expression of the relaxin 2 receptor, RXFP1. We did not detect any significant differences between the tumor stage within examined subtypes. Interestingly, among papillary tissues, the strongest RXFP1 detection was in the papillary adenoma tissue (*n* = 1). Generally, the expression in papillary tumors was significantly weaker than in clear cell tumors (* p ≤ 0.02) (Figure 5). No correlation in expression of RXFP1 and relaxin 2 was noted.

## 3.3. Secreted Proteins

Serum samples were investigated for the level of secreted proteins, which may influence the renal activity of AR and RLN2. The secreted levels of the main activator of AR testosterone were not different between ccRCC and pRCC or based on tumor size, and were in the normal range (males 8.4–28.7 nmol/L, females 0.5–2.6 nmol/L). The serum levels of RLN2 in both tumor types were under the detection limit (≤7.8 pg/mL). Among the interleukins, 70% of pRCC samples but only 45% of ccRCC samples lacked IL-6 secretion (Table 5 and Table 6). The mean values of the pT1 and pT2 male cohorts were approximately 7.5 and 1.7 times higher in pRCC than in ccRCC, respectively (normal range ≤ 5.9 pg/mL). The IL-8 levels in the female pT1 ccRCC population were higher than those in the pT1 pRCC, and in the male pT2 ccRCC populations were lower than in the pT2 pRCC. However, all mean values were in the normal range (≤62 pg/mL) (Table 5).

## 4. Discussion

This study clearly demonstrates differences in AR expression between two main types of RCC—ccRCC and pRCC—and between pT stages of ccRCC tumors. Additional correlations between the expression of three types of AR (AR-FL, AR-V1 and AR-V4) and its potential modulator RLN2 suggest that AR may serve as a promising target for therapy in patients with RCC.

Signaling related to AR is multistage and complex and may affect other processes, such as inflammation, epithelial–mesenchymal transition, cell migration or proliferation, which are crucial for the development and metastasis of tumors.

Extensive analysis has classified RCC as a hormone-related disease [50]; therefore, the association between AR and RCCs has also been a topic of interest in many studies. However, the results have been controversial, with some studies correlating AR expression with low-stage tumor status and good prognosis [7], and others correlating it with poor prognosis [51], as reviewed by Yuan et al. [50]. The majority of the analyses were performed on the most common type of RCC (ccRCC) and knowledge about AR in pRCC is limited. Interestingly, Zhu et al. [6] reported that the AR expression in normal adjacent tissues is higher than that in ccRCC tumors. An analysis performed by Foersh et al. [52] revealed higher expression of AR in pRCC than in ccRCC. In our cohorts, both normal and tumor tissues of pRCC patients revealed higher expression of AR-FL than did normal and tumor tissues of ccRCC patients (Figure 1). This finding implies a molecular difference between ccRCC and pRCC, suggesting an influence of external factors on the whole kidney and/or genetic predispositions to developing certain types of renal cancer, and may support further pathological analysis and study of targeted/hormone therapy.

In many cancers, the expression of AR is related to tumor stage. For example, bladder cancer is characterized by high expression of AR in tumors of low stage and grade and these features positively affect the survival time of patients [12]. Our results support the thesis of Zhao et al. [53] about the protective role of AR in ccRCC. In the analysis based on tumor stage, our cohort revealed a higher expression of AR in pT1 tissues than in pT4 tissues of ccRCC patients. Zhu et al. [6] described similar observations, detecting a negative association of AR expression with pT stage and Fuhrman’s grade. Moreover, our cohort included three metastatic patients. Two of them (with pT2 and pT3 disease) developed pulmonary metastasis and, contrary to the observations of Huang et al. [54] but similar to those of Zhu et al. [6], displayed the weakest expression of AR in primary tumors.

In PCa, the crosstalk between AR signaling and other signaling pathways is well described. Androgen-independent growth of tumor cells can be mediated by the hormone RLN2, which can activate the AR signaling pathway by inducing the formation of the β-catenin–AR complex and its translocation to the nucleus [27,55]. Analysis of the interaction between relaxin and glucocorticoid receptor (GR) in other systems has revealed the ability of relaxin to directly bind and activate GR [56], which can then stimulate AR expression and activity [57].

As previously mentioned, targeted therapies administered to RCC patients may achieve disappointing results due to adaptation of the tumor microenvironment and, consequently, resistance [19]. Recently, Hu et al. [58] described their experiments, in which they introduced relaxin plasmids into hepatic metastatic lesions, using hepatic stellate cell (HSC) targeted nanoparticles. HSCs possess the ability to differentiate into cancer-associated fibroblasts (CAFs). CAFs are involved in the creation and remodeling of the extracellular matrix (ECM), reprogramming of tumor metabolism and creation of a suppressive tumor immune microenvironment, which can lead to chemoresistance [59]. Stimulation of HSCs with relaxin induces an antifibrogenic phenotype of HSCs and impedes the prometastatic ability of CAFs and their properties to modulate the immune milieu in the tumor microenvironment. Additionally, the combination of relaxin and PD-L1 plasmids showed even better results with an improved survival rate and reduced metastases [60,61].

Renoprotective effects of relaxin, including attenuation of fibrosis, have been noted in patients with several diseases, such as dilated cardiomyopathy or age-related renal fibrosis [32,33,34]. In our cohort, relaxin expression was lower in tumors than in normal adjacent tissue. Moreover, even if the level of secreted relaxin was below the detection level, the expression of RXFP1 especially in ccRCC allows us to speculate that the renal tumors can be a target of locally present RLN2 and opens the possibility to initiate the study on any relaxin therapy in renal tumors. We speculate that the potential protective activity of relaxin in the kidneys of RCC patients is age dependent, and in the case of our cohort, this activity was rather low. However, this hypothesis requires further clarification. Furthermore, our data identified a negative association between relaxin expression and tumor growth and a significant positive correlation between relaxin expression and AR expression (r = 0.67, * *p* ≤ 0.002) in the male cohort. These data suggest the possibility of indirect dependency or direct crosstalk between relaxin and AR in renal carcinoma, especially in men with ccRCC.

Our investigations were not limited to AR-FL only. We strengthened the study by analyzing the expression of five SVs (AR-V1, AR-V3, AR-V4, AR-V7 and AR-V12) in patient samples. The exact function of the SVs is not clear. Similar to previously described reports, we found that the expression of AR-FL and all the SVs, except AR-V12, differed between ccRCC tumors of different pT stages with pT1 tumors showing the highest expression and pT4 tumors showing the lowest expression. Additionally, in both pT1 and pT4 but not in pT2 and pT3 tumors, the levels of AR-V7 were increased when compared to those of AR-FL. AR-V12 was not detected in any tissue. Strikingly, in prostate cancer, expression of AR-V7 is considered as a marker limiting treatment or predicting poor prognosis [20,22] or as a constitutively active replacement for AR [62]. Various investigations have reported the role of AR and AR-SV in the regulation of transcription. Many of their targets are similar; however, some of them are unique [63]. By integrating these findings with our results, we can suppose that the products of targets that are present in the tumors of pT1 stage are absent in pT4 tumors. It would be interesting to determine whether therapy with serelaxin or nanoparticles with plasmid relaxin, as described for the HSCs [58], could improve the expression of AR and its SVs in high-stage RCC. Additionally, delivered relaxin could activate the AR signaling pathway by inducing the formation of the β-catenin–AR complex [27,55] and improve its protective activity in tumors and ECM.

Constitutively expressed variants of AR were analyzed mainly in PCa. Cytoplasm-localized AR-V4 and AR-V1 dimerize with AR-FL and AR-V7; however, only binding to androgen-bound AR or AR-V7 induces transfer to the nucleus. Additionally, AR-V1 can enhance AR-FL but weaken AR-V7 transactivation in androgen-independent actions [64]. The presence of AR-V4, AR-V1, AR-FL and AR-V7 suggests that similar crosstalk could take place in patients suffering from ccRCC, placing the SVs as regulators of AR-FL and AR-V7. Moreover, Zahn et al. [64] suggested that by lacking inherent transcriptional activity, AR-V1 acts as an activator of AR-FL in an androgen-independent manner. The presence of SVs in PCa correlates with therapy resistance. Their function in renal carcinoma is not known. The presented results can be the basis for further investigation of the role of AR-FL and AR-SVs in renal carcinoma to determine the effect of presently accepted therapies on the localization and activity of the SVs. This knowledge could help in therapy response prediction.

Cytokines, particularly IL-6, may play an important role in RCC. According to Favaro et al. [44], IL-6 is detectable in the serum of metastatic RCC patients but not in healthy people. Moreover, it is associated not only with the proliferation and invasion but also with the resistance of RCC [45]. We investigated two tumor types: ccRCC (worse prognosis) and pRCC (better prognosis). We could not detect any differences in either examined interleukin (IL-6 and IL-8) between the patients with different tumor stages. However, we showed that 70% of pRCC and only 45% of ccRCC patients lacked detectable levels of IL-6 in serum. This finding is in agreement with the previously described results of Favaro et al. [44], demonstrating the ability of IL-6 in serum to predict poor prognosis in RCC patients. In addition, in vitro analysis has revealed the involvement of IL-6 in the invasion process [33].

## 5. Conclusions

Our results provide deeper insight into the possible roles and crosstalk of AR and RLN 2 in both ccRCC and pRCC. We demonstrate that AR-FL and the constitutive splice variants AR-V1, AR-V3, AR-V4 and AR-V7 are more highly expressed in pRCC than in ccRCC in both tumor and corresponding control tissues. Moreover, the expression is lower in advanced ccRCC tumors than in early-stage ccRCC tumors. The male cohort showed a positive correlation between AR and RLN2 expression. Analysis of secreted cytokines revealed a differential pattern in the two types of RCC.

## 6. Limitations

Our study was performed on human tissues originating from patients with different stages and subtypes of renal carcinoma. We must address several limitations. In the investigations were used a low number of samples. Studies with larger collectives are planned to be performed. The lack of functional correlation analysis should be addressed in the future. For better validation of our results, studies with more samples are planned.

## Figures and Tables

**Table 1:** Tissues samples used in the study.

Tissue (*n*; Tissue/Serum)	Gender (*n*; Tissue/Serum)	pT (*n*; Tissue/Serum)
ccRCC (25/20)	M (19/13)	pT1 (6/6)
		pT2 (5/2)
		pT3 (6/4)
		pT4 (2/1)
	F (6/7)	pT1 (3/5)
		pT2 (1/1)
		pT3 (2/1)
pRCC (10/10)	M (8/7)	adenoma (1/0)
		pT1 (4/4)
		pT2 (2/2)
		pT3 (1/1)
	F (2/3)	pT1 (0/2)
		pT2 (2/1)

M—male, F—female, ccRCC—clear cell renal cell carcinoma, pRCC—papillary renal cell carcinoma, *n*—number of samples.

**Table 2:** Primers used in the study. (*—[46], **—[47], ***—[48], ****—[49]).

Target	Primer	Product Length (bp)
Relaxin	F: TTGCCACAGGAGCTGAAGTT R: TCTGCGGCTTCACTTTGTCT	146
RXFP1 ***	F: AAAAGAGATGATCCTTGCCAAACG R: CCACCCAGATGAATGATGGAGC	299
AR-FL *	F: CAGCCTATTGCGAGAGAGCTG R: GAAAGGATCTTGGGCACTTGC	73
AR-V1	F: AGGGAAAAAGGGCCGAGCTA R: TCCTCCGAGTCTTTAGCAGC	185
AR-V3	F: AAGAGCCGCTGAAGGGAAAC R: AGGCAAGTCAGCCTTTCTTCA	199
AR-V4	F: CTCTCAGCTGCTCATCCACA R: GGTTTTCAAATGCAGCCAGGA	74
AR-V7 ****	F: AAAAGAGCCGCTGAAGGGAA R: GCCAACCCGGAATTTTTCTCC	150
AR-V12 ** (ARv567es)	F: CCAAGGCCTTGCCTGATTGC R: TTGGGCACTTGCACAGAGAT	120
β-Actin	F: ATTGCCGACAGGATGCAGAA R: GCTGATCCACATCTGCTGGAA	150

**Table 3:** AR and SV expression in RCC (mRNA).

ccRCC	AR-FL (*n*)	AR-V1 (*n*)	AR-V3 (*n*)	AR-V4 (*n*)	AR-V7 (*n*)
N > T	56% (14)	56% (14)	52% (13)	64% (16)	48% (12)
N = T	12% (3)	12% (3)	8% (2)	4% (1)	8% (2)
N < T	32% (8)	32% (8)	40% (10)	32% (8)	44% (n1)
**Papillary** **adenoma**	**AR-FL (*n*)**	**AR-V1 (*n*)**	**AR-V3 (*n*)**	**AR-V4 (*n*)**	**AR-V7 (*n*)**
	N < T (1)	N > T (1)	N = T (1)	N > T (1)	N > T (1)
**pRCC**	**AR-FL (*n*)**	**AR-V1 (*n*)**	**AR-V3 (*n*)**	**AR-V4 (*n*)**	**AR-V7 (*n*)**
N > T	44% (4)	11% (1)	44% (4)	56% (5)	56% (5)
N = T	0% (0)	0% (0)	0% (0)	0% (0)	0% (0)
N < T	56% (5)	89% (8)	56% (5)	44% (4)	44% (4)

M—male, F—female, N—normal tissue, T—tumor tissue, RCC—renal cell carcinoma, ccRCC—clear cell RCC, pRCC—papilary RCC, AR—androgen receptor, *n*—number of samples.

**Table 4:** Correlation between RLN2 and AR (AR-FL and AR–SVs) expression in tumor tissues of male ccRCC patients and the whole cohort of pRCC patients. Correlations between RLN2 and all AR variants were calculated with the Spearman test. Significant values are marked with the asterisk *.

		AR-FL	AR-V1	AR-V3	AR-V4	AR-V7
**ccRCC**						
RLN2	r ≤	0.67	0.58	0.20	0.49	0.16
	*p* ≤	* 0.002	* 0.01	0.42	* 0.04	0.52
**pRCC**						
RLN2	r ≤	−0.08	0.07	0.36	0.77	0.45
	*p* ≤	0.84	0.88	0.34	* 0.01	0.23

**Table 5:** Secreted proteins. Testosterone and interleukin levels measured in the serum of ccRCC and pRCC patients are presented as median (min–max), in cases in which a single sample was assigned to a group, single values are shown.

Sex/pT	Testosterone (nmol/L)	IL6 (pg/mL)	IL8 (pg/mL)
**ccRCC**			
M/pT1	15.60 (10.13–20.35)	3 (<2.00–3.21)	12.20 (8.93–25.60)
F/pT1	0.64 (0.27–1.05)	4.20 (<2.00–4.75)	11.85 (<5.00–64.90)
M/pT2	10.69 (6.51–14.87)	3.37 (<2.00–3.37)	7.35 (6.20–8.51)
M/pT3	15.95 (8.39–20.72)	3.715 (<2.00–4.54)	10.9 (8.75–13.2)
F/pT3	0.30	40.70	123
M/pT4	10.50	3.96	35.60
**pRCC**			
M/pT1	11.34 (6.90–15.66)	22.60 (<2.00–41.1)	13.19 (<5.00–18.90)
F/pT1	0.46 (0.41–0.52)	<2.00	6.61 (6.46–6.77)
M/pT2	13.97 (10.47–17.47)	5.79 (<2.00–5.79)	16.66 (8.63–24.70)
F/pT2	<0.24	<2.00	7.24
M/pT3	12.42	<2.00	6.67

M—male, F—female, RCC—renal cell carcinoma, ccRCC—clear cell RCC, pRCC—papillary RCC, n—number of samples, ND—not detected.

**Table 6:** Secreted proteins. Testosterone and interleukin levels measured in the serum of ccRCC and pRCC patients are presented as percentage distribution in the group.

Sex/pT (n)	Testosterone		IL6		IL8		
	Positive % (n)	ND % (n)	Positive % (n)	ND % (n)	Positive % (n)	ND % (n)	
ccRCC							
M/pT1 (6)	30 (6)	0 (0)	20 (4)	10 (2)	30 (6)	0 (0)	
F/pT1 (5)	25 (5)	0 (0)	10 (2)	15 (3)	15 (3)	10 (2)	
M/pT2 (2)	10 (2)	0 (0)	5 (1)	5 (1)	10 (2)	0 (0)	
F/pT2 (1)	5 (1)	0 (0)	0 (0)	5 (1)	5 (1)	0 (0)	
M/pT3 (4)	20 (4)	0 (0)	10 (2)	10 (2)	20 (4)	0 (0)	
F/pT3 (1)	5 (1)	0 (0)	5 (1)	0 (0)	5 (1)	0 (0)	
M/pT4 (1)	5 (1)	0 (0)	5 (1)	0 (0)	5 (1)	0 (0)	
Total 20 (100%)	20/20 (100%)	0/20 (0%)	11/20 (55%)	9/20 (45%)	18/20 (90%)	2/20 (10%)	
pRCC							
M/pT1 (4)	40 (4)	0 (0)	20 (2)	20 (2)	20 (2)	20 (2)	
F/pT1 (2)	20 (2)	0 (0)	0 (0)	20 (2)	20 (2)	0 (0)	
M/pT2 (2)	20 (2)	0 (0)	10 (1)	10 (1)	20 (2)	0 (0)	
F/pT2 (1)	0 (0)	10 (1)	0 (0)	10 (1)	10 (1)	0 (0)	
M/pT3 (1)	10 (1)	0 (0)	0 (0)	10 (1)	10 (1)	0 (0)	
Total 10 (100%)	9/10 (90%)	1/10 (10%)	3/10 (30%)	7/10 (70%)	8/10 (80%)	2/10 (20%)	

M—male, F—female, RCC—renal cell carcinoma, ccRCC—clear cell RCC, pRCC—papillary RCC, *n*—number of samples, ND—not detected.

## Data Availability

The data are not publicly available due to privacy and ethical restrictions.

## Supplementary Materials

The following are available online at https://www.mdpi.com/article/10.3390/life11080731/s1, Figure S1: Summary of online data basis analysis. (a) Expression of AR protein in kidney (3; orange) (https://www.proteinatlas.org/, accessed on 16 July 2021), (b) Expression of AR in normal and tumor tissues (http://gent2.appex.kr/gent2/, accessed on 16 July 2021), (c) Survival probability of RCC patients with low and high expression of AR.
