# Supplementary material for: Differential Expression of the Androgen Receptor, Splice Variants and Relaxin 2 in Renal Cancer
Source: Life (Basel). 2021 Jul 22;11(8):731. doi: 10.3390/life11080731 (PMC8402134; doi:10.3390/life11080731)
Supplement: Supplementary file 1 [file life-11-00731-s001.zip › life-1292267-supplementary.pdf]

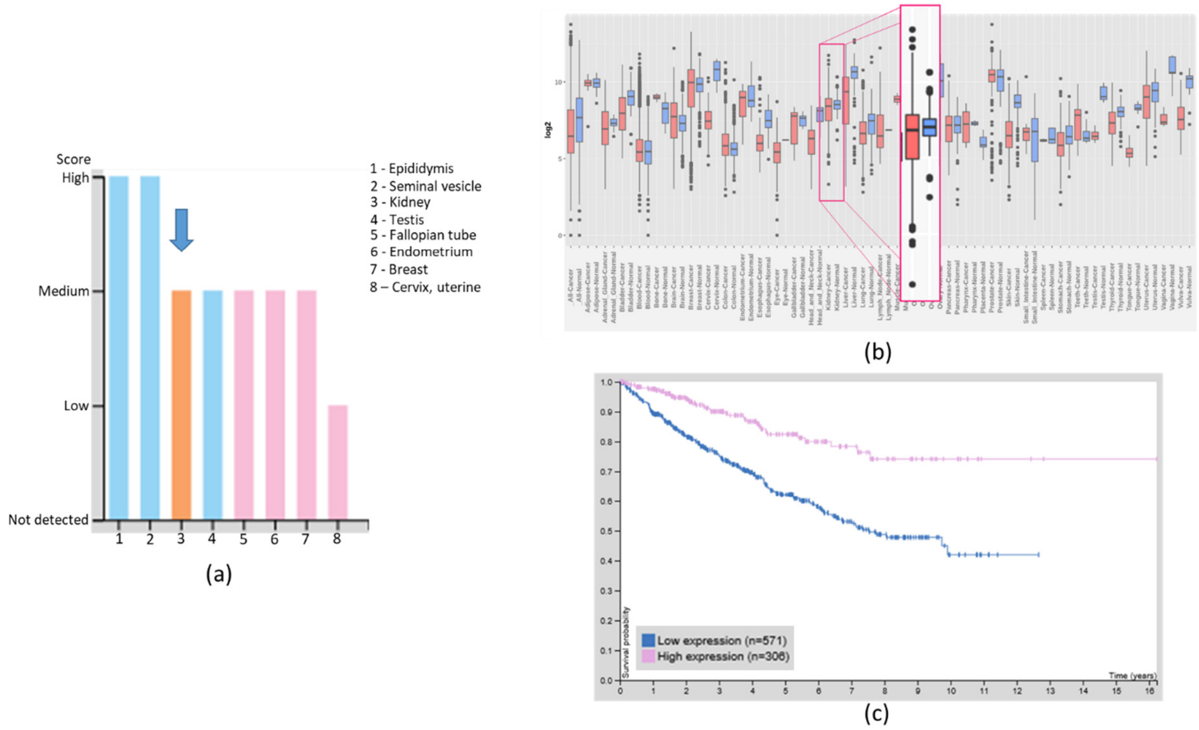

Figure S1. Summary of online databasis analysis. (**a**) Expression of AR protein in kidney (3; orange) (https://www.proteinatlas.org/), (**b**) Expression of AR in normal and tumor tissues (http://gent2.appex.kr/gent2/), (**c**) Survival probability of RCC patients with low and high expression of AR.
